# Mosses Are Better than Leaves of Vascular Plants in Monitoring Atmospheric Heavy Metal Pollution in Urban Areas

**DOI:** 10.3390/ijerph15061105

**Published:** 2018-05-29

**Authors:** Yanbin Jiang, Miao Fan, Ronggui Hu, Jinsong Zhao, Yupeng Wu

**Affiliations:** Key Laboratory of Arable Land Conservation (Middle and Lower Reaches of Yangtze River), Ministry of Agriculture, College of Resources and Environment, Huazhong Agricultural University, Wuhan 430070, China; jiangyanbin@mail.hzau.edu.cn (Y.J.); fanmiao@webmail.hzau.edu.cn (M.F.); rghu@mail.hzau.edu.cn (R.H.); jszhao@mail.hzau.edu.cn (J.Z.)

**Keywords:** atmospheric deposition, moss, tree leaves, anthropogenic factor, contamination factor

## Abstract

Mosses and leaves of vascular plants have been used as bioindicators of environmental contamination by heavy metals originating from various sources. This study aims to compare the metal accumulation capabilities of mosses and vascular species in urban areas and quantify the suitability of different taxa for monitoring airborne heavy metals. One pleurocarpous feather moss species, *Haplocladium angustifolium*, and two evergreen tree species, *Cinnamomum bodinieri* *Osmanthus fragrans*, and substrate soil were sampled in the urban area of different land use types in Wuhan City in China. The concentrations of Ag, As, Cd, Co, Cr, Cu, Mn, Mo, Ni, V, Pb, and Zn in these samples were analyzed by inductively coupled plasma mass spectrometry. The differences of heavy metals concentration in the three species showed that the moss species was considerably more capable of accumulating heavy metals than tree leaves (3 times to 51 times). The accumulated concentration of heavy metals in the moss species depended on the metal species and land use type. The enrichment factors of metals for plants and the correlations of metals in plants with corresponding metals in soil reflected that the accumulated metals in plants stemmed mostly from atmospheric deposition, rather than the substrate soil. Anthropogenic factors, such as traffic emissions from automobile transportation and manufacturing industries, were primarily responsible for the variations in metal pollutants in the atmosphere and subsequently influenced the metal accumulation in the mosses. This study elucidated that the moss species *H. angustifolium* is relatively more suitable than tree leaves of *C. bodinieri* and *O. fragrans* in monitoring heavy metal pollution in urban areas, and currently Wuhan is at a lower contamination level of atmospheric heavy metals than some other cities in China.

## 1. Introduction

Air pollution, which is a consequence of urbanization and industrialization, together with the rapid growth of motorized transportation and population, is one of the most serious threats to the environment and human health in the world, especially in urban areas; moreover, atmospheric heavy metals (e.g., Cr, Cu, Zn, Cd, and Pb) are considered an important group of air pollutants [1,2,3,4,5]. Monitoring and management of air pollution have been conducted for a long time and remain prevalent [6]. At present, the main tools used in assessing air pollution in urban areas include chemical monitoring and biomonitoring. Chemical monitoring is an active technique and can provide information about the levels of different pollutants in the atmosphere. This technique usually requires deploying large numbers of deposition collectors with long-term and short-time intervals of routine sample collection; thus, it is expensive, incapable of detecting extremely heavy metals, and difficult to use for collecting long-time accumulation due to capacity limitation [7]. Biomonitoring methods combined with chemical analyses have been extensively researched and applied in recent years [8,9,10].

Various biological matrices have been used in biomonitoring to assess air quality and atmospheric elements, such as soil crust [11], lichens [1,12,13,14,15], bryophytes [16,17,18,19,20,21,22], tree barks and leaves [10,23,24,25]. Of all these biological groups, lichens and bryophytes (mosses) are more frequently used than the other groups to detect atmospheric metal deposition in terrestrial ecosystems, and they are especially recommended for use in large-scale surveys due to their special morphological structures, accumulation mechanisms, and ecophysiology [7,26]. Mosses and lichens have no cuticle layer and no real roots. They can absorb nutrients, pollutants, and moisture directly from the ambient air and retain them. The analysis of metal content in mosses or lichens reflects the atmospheric metal deposition. These cryptogams are distributed widely throughout the world, and their morphologies do not vary with the seasons; thus, using them has the advantages of low cost and easy application throughout the year; that is, biomonitoring allows simultaneous monitoring and sampling at multiple sites [7,19]. Nevertheless, in comparison with lichens, mosses are more geographically extensive in their distribution, more commonly occur in urban areas, and are more convenient to use by employing passive and active accumulators [17,27]. Mosses act as bioindicators and bioaccumulators of metal deposition in the environment, often by considering the diversity and development of naturally growing bryophytes and moss bags, and have been extensively employed over the past decades in the studies of atmospheric contamination, especially by heavy metals [28,29,30,31]. Mosses, particularly the pleurocarpous feather species, are considered suitable biomonitors for assessing the long-term accumulation of deposited airborne metals because of the aforementioned morphologies, their high cationic exchange capacity, and high surface-to-volume or -weight ratio, which favours the accumulation of pollutants [31]. Vascular plants (trees) are not better indicators for air pollution monitoring than mosses or lichens; however, they are the major plant types found in urban areas with high degrees of pollution, where mosses are rarely distributed; thus, trees have been introduced to monitor heavy metal pollution in recent years [32]. The tree species used as biomonitors for air and soil pollution are diverse along different counties, and most of them are evergreen trees, including needles and broadleaves [9,23,33,34]. Only a few studies have reported the biomonitoring of airborne heavy metal by mosses and trees simultaneously, and tree barks are widely applied [35,36,37].

Wuhan, a city recognized as the political, economic, financial, cultural, educational, and major transportation center in Central China, is located at latitude 29°58′–31°22′ N and longitude 113°41′–115°05′ E, east of the Jianghan Plain, and is at the confluence of the Han River and Yangtze River along the middle reaches of the latter. This city is characterized by a subtropical humid monsoon climate with distinct seasonal divisions. The mean monthly air temperature is from 3 to 28.8 °C, with January being the coldest month. The annual precipitation is from 1100 to 1300 mm, half of which occurs between May and July. The wind speed in the city is not strong, with annual mean wind velocity approximately 1.8 m s^−1^ and no obvious variations among months (http://www.weather.com.cn/). As a major transportation hub, dozens of railways, roads, and expressways pass through Wuhan. The heavy industries, including steel works, are also in development. Chemical monitoring is set in several positions in the city, which provides real-time air quality measurements, including the levels of different pollutants in the atmosphere (e.g., PM_10_, SO_2_, NO_2_, CO, and O_3_; data available at http://hbj.wuhan.gov.cn/). To date, the use of mosses as bioindicators for the study of the dynamics of atmosphere pollution in China has been proven in several studies [21,27,38]. However, whether mosses or vascular species are more applicable in monitoring air pollution has not been determined, especially in rapid-urbanization cities, such as Wuhan. Thus, the aims of this study are (1) to test whether the metal accumulation capabilities are different in mosses and vascular species in the urban area of Wuhan where different eco-functional uses are covered; and (2) to quantify the suitability of moss or tree leaves for monitoring airborne heavy metal pollution.

## 2. Materials and Methods

### 2.1. Sample Collection

**Figure 1:**
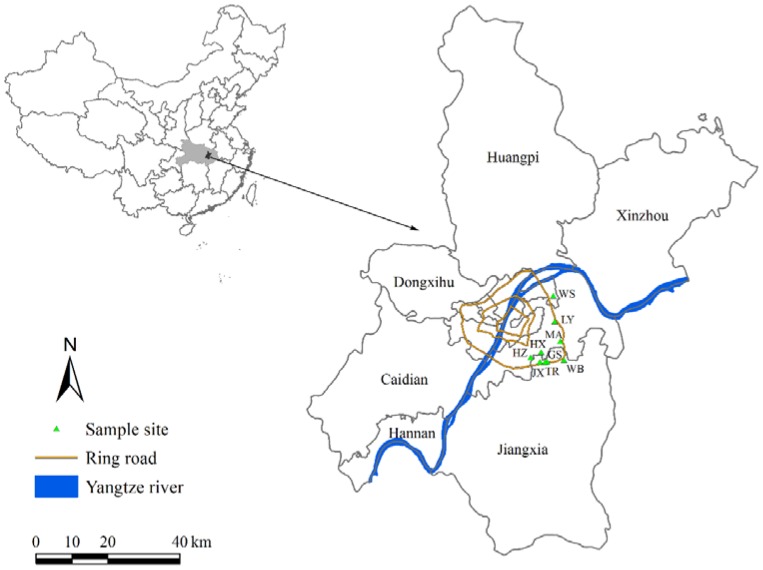
Map of the study area with sampling sites in the urban area of Wuhan.

The study was conducted in the urban area of Wuhan City, Hubei Province, China. Nine sampling sites with different land uses in March 2017 were set in the urban area. These land use types were decided according to the Wuhan urban function zonation (http://gtghj.wuhan.gov.cn/), and included industry, main roadside, university campus, and residential area, which represented different urban eco-functional regions and human activities that would influence the atmospheric pollution. The detailed information of the nine sampling sites is provided in Figure 1 and Table 1.

A moss species (*Haplocladium angustifolium*) and two vascular species (*Cinnamomum bodinieri* and *Osmanthus fragrans*) were used as indicator species. The *H. angustifolium* is the most dominant moss species in Wuhan [39], with creeping main stems and pinnate branching systems. The two vascular species are evergreen broadleaved trees, and are planted as decorative and street trees in the urban area of Wuhan. All three species are widely distributed and easy to collect in the city.

In the open and green vegetated area of each sampling site, three replicates of *H. angustifolium* grow in the ground; nearby, *C. bodinieri*, *O. fragrans* and surface soil (with a depth of 0–5 cm) were collected. For tree species, five leaves in the middle parts of the branches (approximately 2 or 3 years old) from each side (i.e., west, east, south, and north) of a tree were collected and mixed as one sample, three replicates were sampled on three trees. All sampled materials were picked up with a plastic shovel and stored in plastic bags to avoid manual contamination, and then taken back to laboratory for further analysis.

### 2.2. Sample Preparation and Chemical Analysis

Dead material, soil particles, and litter were manually removed for the moss samples. The green or greenish-brown parts of mosses and tree leaves were then cleaned from dust particles with deionized water. These plant and soil samples were dried to a constant weight in a thermostatic drying machine for 48 h at 40 °C. The plant samples were then ground into fine powder in a mill. The soil samples were homogenized with a mortar and pestle after the coarse material was removed using a 2 mm sieve. All these powder samples were kept in clean, dry paper bags. The use of metal equipment was avoided during the operation process to avoid affecting the results of the experimental measurements. Approximately 0.5 g of each plant sample was transferred into a digestion tube and cold digested with 10 mL of mixed acid (HNO_3_:H_2_O_2_ = 4:1), and 0.25 g of each soil sample was digested with 10 mL of mixed acid (HNO_3_:HCl:HF = 3:1:1) for 30 min and then moved to a microwave oven (Mars 6, CEM, Matthews, NC, USA) for enhanced digestion until transparent solutions were obtained. Here we used different mixed acid for digesting plants and soil because there were large amounts of silicate contained in the soil samples that could not be digested completely in mixed acid of HNO_3_ and H_2_O_2_, but could easily be digested in HF.

After cooling, the plant and soil digests were transferred to a 50 mL volumetric flask. The plant was then filled with deionized water to 25 mL and soil to 50 mL. The presence and concentrations of heavy metals (i.e., Ag, As, Cd, Co, Cr, Cu, Mn, Mo, Ni, Pb, V, and Zn) were determined by inductively coupled plasma mass spectrometry (ICP-MS, Flexar LC-NexION 350X, PerkinElmer, Shelton, CT, USA). The concentration of each element in the moss sample was corrected by subtracting blank values. A blank and a plant standard GBW07603 (GSW-2, IGGE, Langfang, China) or a soil standard GBW07403 (GSS-3, IGGE, Langfang, China) were analyzed to check the accuracy and precision of each metal analysis. The recovery percentages of heavy metals were >85% for quantitative analysis. Three replicate measurements per plant and soil sample were performed.

### 2.3. Data Analyses

Concentration values were given as minimum, maximum, mean, and standard deviation (SD). The statistical significance of concentration differences among materials was determined by one-way ANOVA.

Enrichment factors (EF), which determines an element potentially available to an organism from soil, and also evaluates the contribution made by sources other than soil, were calculated for each metal *M* in different plant biomonitors following Equation (1) using Al as the normalizing element and the soil as the reference [40]:

(1) $$\mathrm{EF}=\frac{{\left(\frac{M}{\mathrm{Al}}\right)}_{\mathrm{plant}}}{{\left(\frac{M}{\mathrm{Al}}\right)}_{\mathrm{Soil}}}$$

where M is the concentration of the metal in three plant species under analysis and Al is the concentration of the reference metal chosen, which was determined by ICP-MS. EF ≤ 1 indicates that there is no enrichment of the metal in any of the biomonitors, and values of greater than 1 indicate that there is enrichment of metal M relative to its level in the soil.

The contamination factor (CF) was employed to determine the contamination level of each metal in the study area according to Equation (2):

CF = C_plant_/C_background_ (2)

where C_plant_ is the concentration of the metal in the plant sample in a sample site, C_background_ corresponds the plant samples from clean site (uncontaminated or very slightly contaminated). The scale for interpretation of results would consist of various categories according to the CF values: C1 (CF < 1), none contaminated; C2 (CF: 1 to 2), suspected; C3 (CF: 2 to 3.5), slightly contaminated; C4 (CF: 3.5 and 8), moderated contaminated; C5 (CF: 8 to 27), serious contaminated; C6 (CF > 27), extreme contaminated [40].

Multiple comparisons were performed to determine whether any differences existed between the averages of the materials and sites assumed as polluted and the control region. Pearson’s correlation coefficients (*r*) were calculated to establish the possible correlations between metal concentrations in mosses and soils and among different metals in mosses. Factor analysis with the principal component factoring method based on eigenvalue was employed as an extension of the correlation analysis to clarify the links among metals that tended to have similar origins at the sampling sites and infer possible sources of heavy metals. Dataset was transformed to normal distribution before parametric statistics analyses. All the statistical analyses were performed using the R software.

## 3. Results and Discussion

### 3.1. Comparisons of Trace Element Concentrations in Moss with That in Leaves of Vascular Species

The ranges of metal concentrations in the moss species *H. angustifolium* and in the leaves of the vascular species *C. bodinieri* and *O. fragrans* are displayed in Table 2. The metal concentrations in moss and vascular species differed significantly (*p* < 0.05) and varied at different sampling sites because the minimum, maximum, and SD values varied considerably (e.g., Mn (range: 127–700 μg g^−1^, SD: 189 μg g^−1^). The metal concentrations in mosses were as follows: Mo > Mn > Zn > Pb > Cu > V > Cr > Ni > Co > As > Cd > Ag, with the mean values of Mo, Mn, and Zn being higher than 100 μg g^−1^ and those of Cd and Ag less than 1 μg g^−1^. Of the vascular plants, the interspecies differences in heavy metal concentrations were generally insignificant. The metal concentrations in *C. bodinieri* were as follows: Mn > Mo > Zn > Cu > Pb > V > Cr > Ni > As > Co > Cd > Ag, with only Mn exceeding 100 μg g^−1^ and most metals being less than 1 μg g^−1^. The metal concentrations in *O. fragrans* were as follows: Mo > Mn > Zn > Cu > Pb > V > Ni > Cr > Co > As > Cd > Ag, with Mo and Mn being higher than 100 μg g^−1^ and most metals being less than 1 μg g^−1^. All of these metals in leaves of the two tree species were much lower than those in soil samples. Even for *H. angustifolium*, only the concentration of Ag, Cd, and Zn were higher than those in soil, and the concentration of Cu, Mo and Pb were similar in both *H. angustifolium* and soil samples (Table 2).

The accumulation capabilities of metals by mosses were stronger than those by tree leaves. Table 3 indicates that mosses accumulated heavy metals 4 times to 51 times more than *C. bodinieri* and 3 times to 18 times more than *O. fragrans*. The obviously higher metal concentrations in the moss species than those in both tree leaves observed in this study indicated the metal accumulation capabilities of mosses. A similar trend has been reported in Harjavalta in Finland, where the heavy metal (i.e., Fe, Zn, Cu, Ni, Cd, and Pb) concentrations were ordered as bryophytes > lichens > vascular plants, with the exception of Mn, which followed the order: vascular plants > bryophytes > lichens [37]. Mn concentration was also not consistently higher in mosses than in vascular plants from all sampling sites in our study, that is, the Mn concentrations in *C. bodinieri* and *O. fragrans* from the HZ were higher than those in mosses (Table S1). High mean concentrations of the metals Mn, Zn, Pb, and Cu were also found in moss samples in Istanbul and the Sivas–Tokat motorway in Turkey [20,41] and Kosovo [29]. Of different vascular plants, needle-leaved trees were used more than broad-leaved trees, and pine tree barks were considered more suitable than needles for biomonitoring purposes [42,43], possibly due to their long-time exposure to air pollution.

**Figure 2:**
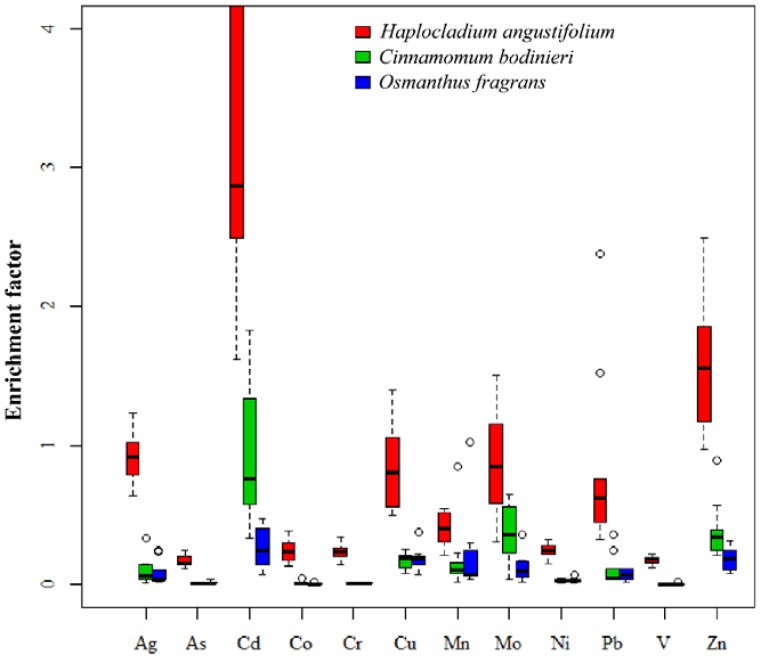
Boxplot of enrichment factors of different heavy metals in three plant samples (*n* = 9) using Al and soil normalization. Circles in the figure indicate the values are outliers.

EFs of all metals in *H. angustifolium* were much higher than those in the two vascular plants, and EFs of Cd, Zn Cu and Mo were highly varied among sampling sites (Figure 2). EFs of *H. angustifolium* showed that the moss species was particularly capable of accumulating Cd, Zn, and Ag, with their EFs being higher than 1. However, the EFs of most metals in the three plants were lower than 1. This can be explained with regard to several aspects, one was that the plant species were incapable of accumulating such metals; secondly, mineral particles trapped by the moss species and tree leaves maybe incompletely solubilized in the case of the digestion method for plants was different from that for soil; and thirdly, these heavy metals were possibly absorbed from sources other than soil. Whether the metals were absorbed from soil or other sources can be roughly determined by the correlation analyses of metals accumulation between plants and soil (Table S2). Vascular plants can uptake metals by roots from soil, absorb atmospheric depositions, and restrict the uptake of toxic elements [37]. Therefore, the heavy metal accumulation of vascular plants was considerably more complicated than that of mosses. Considerable differences in toxic element limits even exist between vascular plant species and ecotypes [44]. Zn and Cu are essential elements to higher plants and are involved in several metabolic processes; thus, they are the least toxic and are often present in higher concentrations [44]. The concentration levels of other toxic elements (i.e., Ag, As, Cd, Cr, and Pb) in the mosses and tree leaves did not exceed those reported as possibly toxic to cultivated soils and plants [45]. Generally, mosses are much more resistant to high levels of toxic elements than vascular plants [31,44].

### 3.2. Differentiation of Metal Accumulation in Moss Species H. angustifolium

**Figure 3:**
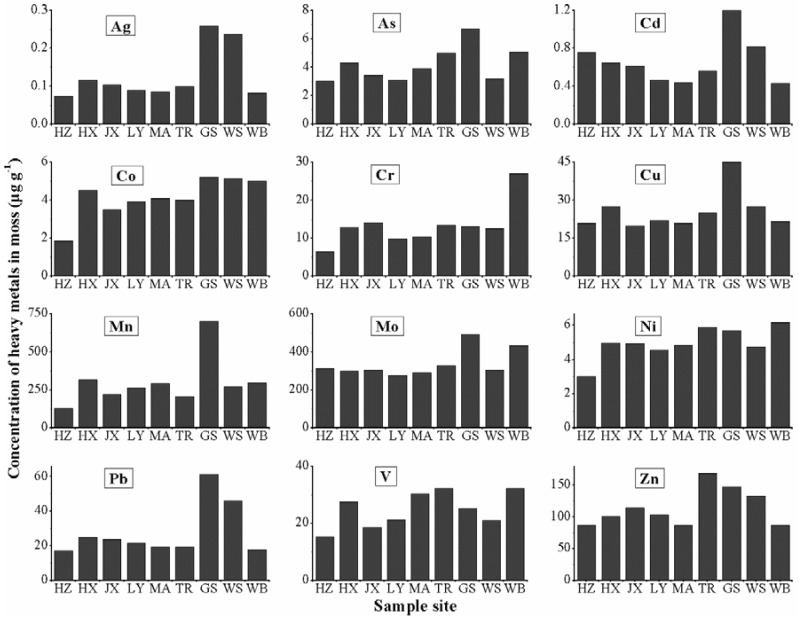
Concentrations of heavy metals in moss species *H. angustifolium* at nine sampling sites in Wuhan.

The heavy metal accumulation in the moss species *H. angustifolium* was much higher than that of tree leaves and close to that of soil, and the EFs of tree leaves were generally much lower than 1. Therefore, *H. angustifolium* was more suitable to monitoring the atmospheric heavy metals in the studied city. Meanwhile, varied metals in the moss species showed different concentrations at different sampling sites. The concentrations of 12 metals in moss samples at each site were obtained to characterize the distribution differences, and they are displayed in Figure 3. The results showed that GS had the highest concentration of most metals, including Ag, As, Cd, Co, Cu, Mn, Mo, Pb, and Zn; TR possessed relatively higher Ni, V, and Zn; WB had high Co, Cr, Ni, and V; and WS had relatively higher Ag, Co, Pb, and Zn. In contrast, HZ was the location with the lowest heavy metal accumulation. TR and GS are main roads or road intersections, where heavy traffic occurs all year long. WB and WS are industrial works in Wuhan. The lower content of heavy metals in the atmosphere than that from automobile transportation may result from the policies of energy saving and emission reduction in recent years. HZ is a university campus located in the far south of the city center with minimal anthropogenic emission, and is surrounded on three sides by clear lakes and backed by green hills with high vegetation cover; thus, HZ is minorly polluted by heavy metals.

Pearson’s correlation analyses were conducted to illustrate the relationships between the same metals in moss species and soil, and the relationships of various metals in the moss species. The results showed that except for the concentrations of Mn and Co in moss species, which were highly significantly correlated with those in soil (Table 4, *p* < 0.05), other metals in the moss species had no significant relations with those in soil. These results proved that the accumulated metals in *H. angustifolium* were mostly not from soil but from the atmosphere. However, Galuszka [42] explained that although mosses do not take up substances directly from soil, soil particulates may strongly influence the chemical composition of mosses by blowing wind and deposition on moss surfaces and then partly dissolve by precipitation, thereby enriching mosses in some elements. Some of the analyzed metals in the moss samples were associated with one another; for example, Ag, Cd, Cu, and Pb had correlation coefficients higher than 0.65, so did Cr, Ni and V (Table 5, *p* < 0.05). The significantly positive correlations between the concentrations of Cd and Pb in mosses have been found in most European countries [29,46]. Significantly positive correlations among Cd, Cu, and Pb were also in agreement with some investigations in China [21,47]. These results indicated that these heavy metals often appear simultaneously and might come from the same pollution sources.

To further analyze the correlation results of metals, factor analysis was executed. Two main factors were extracted and accounted for 80.1% of the total variance of data from the nine sampling sites (Table 6). Factor 1 mainly comprised the metals of Cu, Mn, Pb, As, Ag, and Mo and represented 54.2% of the total variance. The association of these metals with the first factor was likely to be the result of anthropogenic activities, including automobile transportation and industrial emissions [29,48]. Factor 2 explained 25.9% of the total variance and was mainly influenced by high loadings of V, Cr, and Ni. This association was likely to be related to manufacturing industries and heat production [49], such as coal-fired boiler works.

### 3.3. Atmospheric Heavy Metal Pollution Assessing by CF

As the moss species was more suitable to monitoring the atmospheric heavy metals in the studied city, we took the metal concentration in moss samples in different sampling sites to calculate CF, called CF_moss_. For CF_background_, the concentration of same metal in moss sample from the sampling site HZ was used, since this site was relatively much cleaner than other sites and was very slightly contaminated by heavy metals, as we mentioned above. As shown in Table 7, none of the 12 metals have yet reached moderate contamination, indicating an overall low contamination level of atmospheric heavy metals in the city. However, the CF values in some sampling sites, e.g., Cr in WB, Mn in GS and Pb in GS, were higher than 3.5, which means that these sites experienced moderate contamination. The contamination categories determined by moss monitoring in the study was somehow close to the European cities Galicia [40] and Kosovo [29], but lower than some other cities in China, such as Taizhou [28] and Wuxi [21]. This is probably associated with the land use and land cover differences. For example, main industries such as electrical, chemical, pharmaceutical, textile have resulted in high levels of environmental pollution in Taizhou [21]. Although Wuhan is a major transportation center and industries are developing, it is also called “the city of the hundred lakes”, and 40% of the territory is covered by wetlands.

## 4. Conclusions

The concentrations of heavy metals in the moss species *H. angustifolium* were significantly higher than those in the leaves of vascular species *C. bodinieri* and *O. fragrans*, and the EFs performed similarly. Soil substrate exerted a minimal effect on the heavy metal concentrations in the *H. angustifolium*, which indicated that the heavy metals in the mosses were mainly derived from atmospheric deposition. These results indicated that the moss species was more suitable than the tree leaves for monitoring atmospheric heavy metals. Among the 12 studied heavy metals, Mn, Mo, Zn, Cu, and Pb were the metals with the highest concentrations in all the materials analyzed in this study, which implied that the air was most polluted by these metals. The accumulation concentrations of heavy metals in mosses were metal specific and spatially differed, thereby reflecting the local variations in heavy metal deposition. The high concentrations of metals in the sites near main roads and industrial works indicated that anthropogenic factors, such as traffic emission from automobile transportation and manufacturing industries, were primarily responsible for the variations in metal pollutants in the atmosphere. CF values indicated an overall low contamination level of atmospheric heavy metals in Wuhan.

## Figures and Tables

**Table 1:** Sample locations and general characteristics.

Site No.	Location (Abbreviation)	Function and Land Use Description	Longitude/E	Latitude/N	Altitude (m)
1	Huazhong Agricultural University (HZ)	Education, University campus	114°21.682′	30°28.625′	60
2	Hongxiang (HX)	Residential land, residential area	114°23.588′	30°29.285′	58
3	Jinxiu (JX)	Residential land, residential area	114°23.223′	30°27.859′	48
4	Luoyan (LY)	Scenic spot, park nearby East Lake	114°26.633′	30°33.678′	33
5	Ma’anshan Park (MA)	Scenic spot, park nearby East Lake	114°27.139′	30°30.644′	41
6	Third Ring Road (TR)	Transportation, roadside of urban expressway	114°24.093′	30°27.729′	63
7	Guanshan Overpass (GS)	Transportation, roadside of urban overpass	114°24.439′	30°27.805′	59
8	Wuhan Steel Works (WS)	Industry, manufacturing industry of iron and steel	114°26.638′	30°37.481′	38
9	Wuhan Boiler Works (WB)	Industry, manufacturing industry of boilers	114°27.456′	30°27.748′	39

**Table 2:** Descriptive statistics of element concentrations (μg g^−1^) in moss, tree leaves and soil.

Sample	Value (μg g^−1^)	Ag	As	Cd	Co	Cr	Cu	Mn	Mo	Ni	Pb	V	Zn
*H. angustifolium*	Minimum	0.074	3.04	0.433	1.85	6.37	19.9	127	277	2.99	17.3	15.3	86.4
	Maximum	0.258	6.69	1.20	5.22	27.0	44.9	700	491	6.17	61.3	32.2	169
	Mean	0.127	4.20	0.660	4.14	13.3	25.6	300	338	4.97	28.0	24.8	114
	SD	0.073	1.33	0.27	1.17	6.67	8.94	189	79.2	1.05	16.6	6.39	31.1
	Diff	a	a	a	a	a	a	a	a	a	a	a	a
*C. bodinieri*	Minimum	0.004	0.156	0.025	0.047	0.277	3.54	39.3	17.7	0.326	1.179	0.306	8.47
	Maximum	0.021	0.871	0.092	0.345	1.29	7.41	393	75.7	0.864	3.738	2.553	16.9
	Mean	0.008	0.317	0.050	0.108	0.579	5.23	120	41.1	0.549	2.361	0.735	13.4
	SD	0.006	0.222	0.023	0.091	0.339	1.35	122	17.9	0.185	0.940	0.695	3.32
	Diff	b	b	b	b	b	b	b	b	b	b	b	b
*O. fragrans*	Minimum	0.002	0.111	0.090	0.120	0.357	3.96	13.5	42.9	0.316	1.52	0.369	17.5
	Maximum	0.018	0.318	0.358	0.330	0.759	7.17	223	251	0.976	10.6	0.830	40.9
	Mean	0.009	0.196	0.182	0.224	0.538	5.14	107	144	0.562	3.59	0.575	27.1
	SD	0.005	0.079	0.101	0.057	0.130	1.04	75.0	71.4	0.202	3.09	0.137	7.57
	Diff	b	b	c	b	b	b	b	c	b	b	b	b
Soil	Minimum	0.064	21.5	0.113	12.5	42.4	25.1	386	329	16.4	28.0	102	55.9
	Maximum	0.217	23.8	0.301	19.0	54.7	32.0	1035	549	21.2	41.4	138	81.6
	Mean	0.107	22.7	0.185	16.4	49.3	28.0	667	387	18.4	32.7	124	67.8
	SD	0.047	0.876	0.056	2.14	3.56	2.54	190	74	1.43	4.51	10.9	9.69
	Diff	a	c	c	c	c	a	c	a	c	a	c	c

Note: SD: standard deviation; Different letters of a, b and c in Diff in the same column indicate significant differences at *p* < 0.05 among materials.

**Table 3:** Ratios (mean ± SD) of heavy metals concentration in moss to those in tree leaves.

Metal	*H. angustifolium*/*C. bodinieri*	*H. angustifolium*/*O. fragrans*
Ag	19.8 ± 9.79	46.0 ± 18.6
As	17.4 ± 9.89	25.0 ± 11.8
Cd	15.3 ± 7.76	5.05 ± 3.57
Co	51.3 ± 25.2	20.0 ± 7.25
Cr	29.9 ± 21.3	27.8 ± 14.8
Cu	5.17 ± 1.97	5.27 ± 1.18
Mn	4.08 ± 2.18	6.54 ± 5.92
Mo	10.4 ± 6.62	3.30 ± 2.20
Ni	10.3 ± 4.75	10.7 ± 4.05
Pb	13.6 ± 8.58	12.3 ± 10.4
V	48.5 ± 28.1	48.3 ± 19.9
Zn	9.38 ± 4.94	4.78 ± 1.35

**Table 4:** Pearson’s correlations of metals accumulation between soil and moss based on nine sampling sites.

Metal	Ag	As	Cd	Co	Cr	Cu	Mn	Mo	Ni	Pb	V	Zn
Coefficient (*r*)	0.332	−0.567	0.384	**0.707**	0.168	0.040	**0.941**	0.101	0.218	−0.190	−0.016	−0.042
*p*	0.384	0.112	0.307	**0.033**	0.665	0.919	**0.000**	0.797	0.573	0.624	0.967	0.916

**Table 5:** Coefficients of Pearson’s correlation among accumulated metals in moss based on nine sampling sites.

Metal	Ag	As	Cd	Co	Cr	Cu	Mn	Mo	Ni	Pb	V
As	0.340										
Cd	0.753 *	0.218									
Co	0.578	0.536	−0.048								
Cr	0.217	0.591	−0.231	0.778 *							
Cu	0.842 **	0.642	0.767 *	0.471	0.153						
Mn	0.659	0.665	0.220	0.848 **	0.551	0.700 *					
Mo	0.381	0.811 **	0.399	0.320	0.500	0.610	0.525				
Ni	0.351	0.721 *	−0.162	0.890 **	0.897 **	0.353	0.715 *	0.456			
Pb	0.983 **	0.302	0.774 *	0.524	0.148	0.843 **	0.680 *	0.390	0.282		
V	0.064	0.714 *	−0.373	0.727 *	0.700 **	0.225	0.551	0.324	0.835 **	−0.034	
Zn	0.674 *	0.402	0.537	0.395	0.203	0.634	0.314	0.255	0.457	0.610	0.190

Note: * *p* < 0.05 level; ** *p* < 0.01 level.

**Table 6:** Factor analysis of metals in moss samples.

Metal	Factor 1	Factor 2
Ag	0.818	−0.440
As	0.854	0.310
Cd	0.712	−0.659
Co	0.754	0.381
Cr	0.310	0.804
Cu	0.924	−0.312
Mn	0.912	−0.095
Mo	0.811	0.178
Ni	0.634	0.724
Pb	0.855	−0.461
V	0.323	0.834
Zn	0.593	−0.127
Variance (%)	54.2	25.9

**Table 7:** The contamination factor (CF) and different categories of contamination (as defined by the mean CF) for each of the heavy metal studied in Wuhan.

Metal	Ag	As	Cd	Co	Cr	Cu	Mn	Mo	Ni	Pb	V	Zn
HZ	1.00	1.00	1.00	1.00	1.00	1.00	1.00	2.47	1.00	1.00	1.00	1.00
HX	1.58	1.43	0.85	3.54	2.00	1.31	2.49	2.35	1.66	1.44	1.80	1.17
JX	1.40	1.14	0.81	1.89	2.22	0.95	1.74	2.41	1.64	1.38	1.21	1.32
LY	1.20	1.02	0.62	2.13	1.55	1.05	2.09	2.18	1.52	1.26	1.39	1.19
MA	1.15	1.28	0.58	2.21	1.64	1.00	2.29	2.29	1.61	1.13	1.98	1.01
TR	1.33	1.65	0.74	2.17	2.10	1.19	1.62	2.58	1.96	1.13	2.11	1.95
GS	3.47	2.20	1.58	2.83	2.05	2.14	5.51	3.86	1.90	3.55	1.64	1.70
WS	3.19	1.04	1.08	2.79	1.96	1.31	2.13	2.41	1.58	2.66	1.37	1.54
WB	1.11	1.67	0.57	2.71	4.24	1.03	2.33	3.40	2.06	1.04	2.11	1.00
Mean CF	3.47	2.20	1.58	3.54	4.24	2.14	5.51	3.86	2.06	3.55	2.11	1.95
Category	C2	C2	C1	C3	C3	C2	C3	C2	C2	C2	C2	C2

## Supplementary Materials

The following are available online at http://www.mdpi.com/1660-4601/15/6/1105/s1, Table S1: Element concentrations (μg g^−1^) of three replicates in moss, tree leaves and soil in nine sampling sites, Table S2: Pearson’s correlations of metals accumulation between plants and soil based on nine sampling sites.

## References

[1] Ng O.H., Tan B.C., Obbard J.P. (2006). Lichens as bioindicators of atmospheric heavy metal pollution in Singapore. Environ. Monit. Assess..

[2] Kampa M., Castanas E. (2008). Human health effects of air pollution. Environ. Pollut..

[3] Rai P.K. (2016). Impacts of particulate matter pollution on plants: Implications for environmental biomonitoring. Ecotoxicol. Environ. Saf..

[4] Nagajyoti P.C., Lee K.D., Sreekanth T.V.M. (2010). Heavy metals, occurrence and toxicity for plants: A review. Environ. Chem. Lett..

[5] Li Y.X., Wang Y., Rui X., Li Y.X., Li Y., Wang H.Z., Zuo J., Tong Y.D. (2017). Sources of atmospheric pollution: A bibliometric analysis. Scientometrics.

[6] Ram S.S., Majumder S., Chaudhuri P., Chanda S., Santra S.C., Chakraborty A., Sudarshan M. (2015). A review on air pollution monitoring and management using plants with special reference to foliar dust adsorption and physiological stress responses. Crit. Rev. Environ. Sci. Technol..

[7] Harmens H., Norris D.A., Koerber G.R., Buse A., Steinnes E., Ruhling A. (2007). Temporal trends in the concentration of arsenic, chromium, copper, iron, nickel, vanadium and zinc in mosses across Europe. Atmos. Environ..

[8] Stankovic S., Kalaba P., Stankovic A.R. (2014). Biota as toxic metal indicators. Environ. Chem. Lett..

[9] Ayodele J.T., Ahmed A. (2001). Monitoring air pollution in Kano municipality by chemical analysis of Scots Pine (*Pinus sylvestris* L.) needles for sulphur content. Environmentalist.

[10] Brignole D., Drava G., Minganti V., Giordani P., Samson R., Vieira J., Pinho P., Branquinho C. (2018). Chemical and magnetic analyses on tree bark as an effective tool for biomonitoring: A case study in Lisbon (Portugal). Chemosphere.

[11] Wojtun B., Samecka-Cymerman A., Kolon K., Kempers A.J., Skrzypek G. (2013). Metals in some dominant vascular plants, mosses, lichens, algae, and the biological soil crust in various types of terrestrial tundra, SW Spitsbergen, Norway. Polar Biol..

[12] Paoli L., Munzi S., Guttova A., Senko D., Sardella G., Loppi S. (2015). Lichens as suitable indicators of the biological effects of atmospheric pollutants around a municipal solid waste incinerator (S Italy). Ecol. Indic..

[13] Aprile G.G., Di Salvatore M., Carratu G., Mingo A., Carafa A.M. (2010). Comparison of the suitability of two lichen species and one higher plant for monitoring airborne heavy metals. Environ. Monit. Assess..

[14] Naeth M.A., Wilkinson S.R. (2008). Lichens as biomonitors of air quality around a diamond mine, Northwest Territories, Canada. J. Environ. Qual..

[15] Scerbo R., Ristori T., Possenti L., Lampugnani L., Barale R., Barghigiani C. (2002). Lichen (*Xanthoria parietina*) biomonitoring of trace element contamination and air quality assessment in Pisa Province (Tuscany, Italy). Sci. Total Environ..

[16] Coskun M., Steinnes E., Coskun M., Cayir A. (2009). Comparison of epigeic moss (*Hypnum cupressiforme*) and lichen (*Cladonia rangiformis*) as biomonitor species of atmospheric metal deposition. Bull. Environ. Contam. Toxicol..

[17] Pesch R., Schroeder W. (2006). Mosses as bioindicators for metal accumulation: Statistical aggregation of measurement data to exposure indices. Ecol. Indic..

[18] Pata I.M.C., Balan C.D., Pata S.M., Macoveanu M. (2009). Passive biomonitoring of atmospheric pollution with heavy metals using native Epigeic moss. Environ. Eng. Manag. J..

[19] Bargagli R., Monaci F., Borghini F., Bravi F., Agnorelli C. (2002). Mosses and lichens as biomonitors of trace metals. A comparison study on *Hypnum cupressiforme* and *Parmelia caperata* in a former mining district in Italy. Environ. Pollut..

[20] Icel Y., Cobanoglu G. (2009). Biomonitoring of atmospheric heavy metal pollution using lichens and mosses in the city of Istanbul, Turkey. Fresen. Environ. Bull..

[21] Zhou X.L., Chen Q., Liu C., Fang Y.M. (2017). Using moss to assess airborne heavy metal pollution in Taizhou, China. Int. J. Environ. Res. Public Health.

[22] Wang S.Q., Zhang Z.H., Wang Z.H. (2015). Bryophyte communities as biomonitors of environmental factors in the Goujiang karst bauxite, southwestern China. Sci. Total Environ..

[23] Dogan Y., Ugulu I., Baslar S. (2010). Turkish red pine as a biomonitor: A comparative study of the accumulation of trace elements in the needles and bark. Ekoloji.

[24] Sun F.F., Wen D.Z., Kuang Y.W., Li J.O., Li J.L., Zuo W.D. (2010). Concentrations of heavy metals and polycyclic aromatic hydrocarbons in needles of Masson pine (*Pinus massoniana* L.) growing nearby different industrial sources. J. Environ. Sci. China.

[25] Samecka-Cymerman A., Kolon K., Kempers A.J. (2008). A preliminary investigation in using *Pohlia nutans* and *Larix decidua* as biomonitors of air pollution by the coke industry in Walbrzych (SW Poland). Pol. J. Environ. Stud..

[26] Szczepaniak K., Biziuk M. (2003). Aspects of the biomonitoring studies using mosses and lichens as indicators of metal pollution. Environ. Res..

[27] Sun S.Q., Wang D.Y., He M., Zhang C. (2009). Monitoring of atmospheric heavy metal deposition in Chongqing, China—Based on moss bag technique. Environ. Monit. Assess..

[28] Yan Y., Zhang Q., Wang G.G., Fang Y.M. (2016). Atmospheric deposition of heavy metals in Wuxi, China: estimation based on native moss analysis. Environ. Monit. Assess..

[29] Maxhuni A., Lazo P., Kane S., Qarri F., Marku E., Harmens H. (2016). First survey of atmospheric heavy metal deposition in Kosovo using moss biomonitoring. Environ. Sci. Pollut. Res..

[30] Rasmussen L. (1977). Epiphytic bryophytes as indicators of changes in background levels of airborne metals from 1951–1975. Environ. Pollut..

[31] Tyler G. (1990). Bryophytes and heavy metals: A literature review. Bot. J. Linn. Soc..

[32] Sawidis T., Marnasidis A., Zachariadis G., Stratis J. (1995). A study of air pollution with heavy metals in Thessaloniki City (Greece) using trees as biological indicators. Arch. Environ. Contam. Toxicol..

[33] Sawidis T., Krystallidis P., Veros D., Chettri M. (2012). A study of air pollution with heavy metals in Athens City and Attica basin using evergreen trees as biological indicators. Biol. Trace Elem. Res..

[34] Dongarra G., Sabatino G., Triscari M., Varrica D. (2003). The effects of anthropogenic particulate emissions on roadway dust and *Nerium oleander* leaves in Messina (Sicily, Italy). J. Environ. Monit..

[35] Piraga D., Tabors G., Nikodemus O., Zigure Z., Brumelis G. (2017). Current content of selected pollutants in moss, humus, soil and bark and long-term radial growth of pine trees in the Mezaparks forest in Riga. Environ. Sci. Pollut. Res..

[36] Tsikritzis L.I., Ganatsios S.S., Duliu O.G., Sawidis T.D. (2003). Natural and artificial radionuclides distribution in some lichens, mosses, and trees in the vicinity of lignite power plants from West Macedonia, Greece. J. Trace Microprobe Tech..

[37] Salemaa M., Derome J., Helmisaari H.S., Nieminen T., Vanha-Majamaa I. (2004). Element accumulation in boreal bryophytes, lichens and vascular plants exposed to heavy metal and sulfur deposition in Finland. Sci. Total Environ..

[38] Cao T., Wang M., An L., Yu Y.H., Lou Y.X., Guo S.L., Zuo B.R., Liu Y., Wu J.M., Cao Y. (2009). Air quality for metals and sulfur in Shanghai, China, determined with moss bags. Environ. Pollut..

[39] Fan M., Wu Y., Hu R., Jiang Y. (2017). Diversity and distribution of bryophytes and their relationship with environmental factors in Wuhan. Plant Sci. J..

[40] Fernández J.A., Carballeira A. (2001). A comparison of indigenous mosses and topsoils for use in monitoring atmospheric heavy metal deposition in Galicia (northwest Spain). Environ. Pollut..

[41] Mendil D., Celik F., Tuzen M., Soylak M. (2009). Assessment of trace metal levels in some moss and lichen samples collected from near the motorway in Turkey. J. Hazard. Mater..

[42] Galuszka A. (2005). The chemistry of soils, rocks and plant bioindicators in three ecosystems of the Holy Cross Mountains, Poland. Environ. Monit. Assess..

[43] Sawidis T., Breuste J., Mitrovic M., Pavlovic P., Tsigaridas K. (2011). Trees as bioindicator of heavy metal pollution in three European cities. Environ. Pollut..

[44] Påhlsson A.-M.B. (1989). Toxicity of heavy metals (Zn, Cu, Cd, Pb) to vascular plants. A literature review. Water Air Soil Pollut..

[45] Gough L.P., Shacklette H.T., Case A.A. (1979). Element concentrations toxic to plants, animals, and man. Geol. Surv. Bull..

[46] Harmens H., Ilyin I., Mills G., Aboal J.R., Alber R., Blum O., Coskun M., De Temmerman L., Fernandez J.A., Figueira R. (2012). Country-specific correlations across Europe between modelled atmospheric cadmium and lead deposition and concentrations in mosses. Environ. Pollut..

[47] Liu C., Zhou P., Fang Y.M. (2016). Monitoring Airborne Heavy Metal Using Mosses in the City of Xuzhou, China. Bull. Environ. Contam. Toxicol..

[48] Han Y.M., Du P.X., Cao J.J., Posmentier E.S. (2006). Multivariate analysis of heavy metal contamination in urban dusts of Xi’an, Central China. Sci. Total Environ..

[49] Harmens H., Norris D.A., Koerber G.R., Buse A., Steinnes E., Ruhling A. (2008). Temporal trends (1990–2000) in the concentration of cadmium, lead and mercury in mosses across Europe. Environ. Pollut..

