# Supplementary material for: Mosses Are Better than Leaves of Vascular Plants in Monitoring Atmospheric Heavy Metal Pollution in Urban Areas
Source: Int J Environ Res Public Health. 2018 May 29;15(6):1105. doi: 10.3390/ijerph15061105 (PMC6025423; doi:10.3390/ijerph15061105)
Supplement: Supplementary file 1 [file ijerph-15-01105-s001.zip › ijerph-293855-SI.pdf]

Table S1. Element concentrations (μg g^{-1}) of three replicates in moss, tree leaves and soil in nine sampling sites

Sample sites	Material	Replicate	Al	Ag	As	Cd	Co	Cr	Cu	Mn	Mo	Ni	Pb	V	Zn
HZ	*H. angustifolium*	1	8658	0.079	3.08	0.822	1.89	6.73	21.75	131.13	342.33	3.18	17.91	15.76	88.26
HZ	*H. angustifolium*	2	7074	0.070	2.99	0.695	1.81	6.00	20.20	123.20	286.73	2.81	16.65	14.80	84.57
HZ	*H. angustifolium*	3	7866	0.074	3.04	0.758	1.85	6.37	20.97	127.17	314.53	2.99	17.28	15.28	86.41
HX	*H. angustifolium*	1	20710	0.105	3.94	0.599	4.69	12.67	25.91	244.58	262.94	4.93	19.83	26.52	98.53
HX	*H. angustifolium*	2	19161	0.129	4.72	0.697	4.38	12.85	29.09	388.90	334.93	5.00	30.03	28.41	102.92
HX	*H. angustifolium*	3	19935	0.117	4.33	0.648	4.53	12.76	27.50	316.74	298.94	4.96	24.93	27.46	100.72
JX	*H. angustifolium*	1	16389	0.099	3.88	0.639	4.10	14.66	21.38	239.66	317.08	5.41	20.98	20.71	120.80
JX	*H. angustifolium*	2	17609	0.113	3.82	0.633	3.93	15.56	20.56	250.96	317.56	5.33	29.85	21.37	112.15
JX	*H. angustifolium*	3	8421	0.100	2.66	0.578	2.45	12.17	17.84	174.86	282.86	4.02	20.76	13.49	110.46
LY	*H. angustifolium*	1	15157	0.106	3.24	0.503	3.97	11.48	26.58	270.67	281.15	4.56	28.91	23.28	123.94
LY	*H. angustifolium*	2	15528	0.086	2.95	0.434	3.71	9.09	19.89	260.30	282.86	4.33	19.06	19.90	91.95
LY	*H. angustifolium*	3	16270	0.075	3.11	0.462	4.12	9.00	19.54	265.34	267.11	4.77	17.11	20.57	92.23
MA	*H. angustifolium*	1	15962	0.086	3.55	0.420	3.90	9.95	18.99	276.88	276.12	4.55	18.29	25.23	82.72
MA	*H. angustifolium*	2	17092	0.085	3.88	0.441	4.09	10.45	20.90	291.26	291.20	4.82	19.50	30.33	86.96
MA	*H. angustifolium*	3	16527	0.085	3.72	0.431	3.99	10.20	19.95	284.07	283.66	4.68	18.90	27.78	84.84
TR	*H. angustifolium*	1	21808	0.100	4.78	0.556	3.91	13.87	26.71	204.90	298.79	5.29	18.61	33.89	160.11
TR	*H. angustifolium*	2	24193	0.099	5.27	0.566	4.06	13.49	24.73	208.57	333.01	7.14	20.79	33.03	181.70
TR	*H. angustifolium*	3	19973	0.098	5.00	0.564	4.06	12.79	23.49	205.54	352.02	5.20	19.11	29.61	163.71
GS	*H. angustifolium*	1	16400	0.250	6.31	1.16	4.94	12.03	44.50	663.08	466.32	5.51	59.53	23.51	144.39
GS	*H. angustifolium*	2	17985	0.250	6.28	1.16	5.43	12.91	44.15	730.82	479.86	5.59	58.29	25.84	144.86
GS	*H. angustifolium*	3	17855	0.275	7.50	1.271	5.28	14.15	46.19	707.58	527.75	5.93	66.11	25.82	152.52
WS	*H. angustifolium*	1	9270	0.205	3.09	0.862	8.89	16.70	34.04	265.09	342.57	4.94	44.16	21.36	112.61
WS	*H. angustifolium*	2	9455	0.224	3.10	0.846	2.93	9.69	24.36	254.50	242.37	4.34	46.94	17.84	123.09

WS	*H. angustifolium*	3	14460	0.283	3.34	0.755	3.63	11.12	24.11	292.66	333.05	4.94	46.83	23.72	164.05
WB	*H. angustifolium*	1	20084	0.085	4.47	0.421	4.76	17.65	20.89	261.29	384.72	5.77	17.13	28.18	82.98
WB	*H. angustifolium*	2	20067	0.080	4.46	0.420	4.81	18.34	21.12	261.13	408.57	6.09	17.41	29.14	86.26
WB	*H. angustifolium*	3	21592	0.083	6.32	0.458	5.43	45.03	22.59	367.70	503.35	6.64	19.27	39.30	90.68
HZ	*C. bodinieri*	1	279	0.012	0.337	0.089	0.099	0.450	5.72	226.92	34.11	0.745	2.25	0.506	15.98
HZ	*C. bodinieri*	2	231	0.005	0.272	0.047	0.075	0.387	3.53	292.40	95.55	0.707	1.82	0.443	13.34
HZ	*C. bodinieri*	3	202	0.006	0.271	0.089	0.058	0.429	3.75	219.71	97.47	0.758	3.92	0.375	12.74
HX	*C. bodinieri*	1	732	0.006	0.937	0.040	0.350	1.163	4.87	50.96	45.84	0.572	2.59	2.285	15.14
HX	*C. bodinieri*	2	1305	0.010	0.888	0.054	0.426	1.568	5.84	63.89	54.83	0.616	3.14	3.256	14.98
HX	*C. bodinieri*	3	823	0.010	0.789	0.183	0.261	1.139	5.94	52.61	38.04	0.537	3.20	2.120	15.49
JX	*C. bodinieri*	1	327	0.030	0.316	0.059	0.103	0.476	4.56	242.99	35.84	0.817	3.38	0.524	15.35
JX	*C. bodinieri*	2	342	0.015	0.479	0.055	0.101	0.808	4.89	91.50	30.81	0.647	3.22	0.764	14.18
JX	*C. bodinieri*	3	392	0.018	0.500	0.085	0.129	0.787	6.53	74.71	44.37	0.540	4.12	0.855	16.79
LY	*C. bodinieri*	1	297	0.004	0.235	0.045	0.091	1.018	5.32	44.76	51.17	0.546	4.41	0.690	13.79
LY	*C. bodinieri*	2	276	0.006	0.220	0.045	0.086	0.889	6.02	52.06	54.07	0.558	3.15	0.614	16.74
LY	*C. bodinieri*	3	349	0.003	0.224	0.044	0.110	0.969	4.94	45.84	55.61	0.625	3.65	0.826	14.79
MA	*C. bodinieri*	1	225	0.004	0.237	0.030	0.064	0.391	5.99	76.19	54.77	0.484	1.43	0.509	15.85
MA	*C. bodinieri*	2	281	0.002	0.194	0.037	0.107	0.551	8.60	26.47	41.73	0.372	1.35	0.683	17.46
MA	*C. bodinieri*	3	190	0.007	0.217	0.036	0.058	0.388	6.55	107.06	52.95	0.544	2.37	0.423	17.53
TR	*C. bodinieri*	1	4.00	0.003	0.003	0.002	0.001	0.001	0.091	1.69	1.00	0.015	0.067	0.010	0.095
TR	*C. bodinieri*	2	236	0.008	0.250	0.036	0.078	0.441	5.36	78.16	28.94	0.516	1.86	0.490	11.41
TR	*C. bodinieri*	3	196	0.006	0.216	0.037	0.062	0.390	5.40	38.19	23.26	0.448	1.86	0.419	13.89
GS	*C. bodinieri*	1	250	0.005	0.260	0.044	0.076	0.389	4.40	448.08	19.28	0.904	2.07	0.474	9.28
GS	*C. bodinieri*	2	235	0.008	0.221	0.037	0.073	0.431	5.08	373.26	23.11	0.882	1.97	0.447	9.40
GS	*C. bodinieri*	3	209	0.007	0.222	0.037	0.072	0.378	5.05	356.69	23.76	0.804	1.70	0.424	10.05
WS	*C. bodinieri*	1	88	0.004	0.111	0.024	0.067	0.237	9.18	48.36	35.74	0.322	1.00	0.258	18.06

WS	*C. bodinieri*	2	165	0.013	0.247	0.054	0.076	0.385	7.99	47.21	47.49	0.339	2.28	0.464	15.70
WS	*C. bodinieri*	3	232	0.021	0.382	0.038	0.093	0.517	5.05	41.29	38.04	0.427	3.42	0.628	16.44
WB	*C. bodinieri*	1	138	0.002	0.088	0.036	0.051	0.269	4.62	33.55	25.25	0.289	0.76	0.293	10.32
WB	*C. bodinieri*	2	216	0.007	0.271	0.035	0.074	0.409	2.57	62.30	24.75	0.388	1.53	0.568	9.00
WB	*C. bodinieri*	3	255	0.003	0.182	0.023	0.071	0.384	3.44	47.57	32.02	0.419	1.25	0.499	9.35
HZ	*O. fragrans*	1	379	0.006	0.239	0.189	0.375	0.576	4.27	170.11	71.19	0.385	2.45	0.624	38.73
HZ	*O. fragrans*	2	503	0.005	0.230	0.291	0.403	0.843	2.80	151.79	101.74	0.600	2.59	0.822	35.26
HZ	*O. fragrans*	3	207	0.023	0.126	0.271	0.213	0.455	5.95	292.80	235.61	0.313	3.06	0.399	48.64
HX	*O. fragrans*	1	397	0.017	0.250	0.115	0.249	0.903	4.76	35.05	109.91	1.057	25.96	0.939	36.95
HX	*O. fragrans*	2	259	0.014	0.250	0.093	0.205	0.697	4.01	27.42	69.37	0.236	3.00	0.791	13.22
HX	*O. fragrans*	3	258	0.016	0.253	0.099	0.206	0.678	3.94	42.18	84.00	0.289	2.70	0.762	13.66
JX	*O. fragrans*	1	257	0.009	0.262	0.097	0.214	0.407	5.32	118.09	152.53	0.345	2.42	0.519	23.92
JX	*O. fragrans*	2	250	0.013	0.346	0.089	0.217	0.585	5.86	67.98	199.66	0.687	3.38	0.546	23.92
JX	*O. fragrans*	3	325	0.014	0.346	0.131	0.226	0.548	5.84	34.29	259.86	0.589	2.92	0.571	24.80
LY	*O. fragrans*	1	185	0.003	0.105	0.129	0.112	0.426	5.53	13.34	170.42	0.442	1.44	0.563	18.54
LY	*O. fragrans*	2	161	0.002	0.117	0.137	0.128	0.450	4.85	13.64	180.54	0.222	1.59	0.500	16.45
LY	*O. fragrans*	3	173	0.002	0.111	0.133	0.120	0.438	5.19	13.49	175.48	0.332	1.52	0.531	17.49
MA	*O. fragrans*	1	184	0.007	0.150	0.591	0.277	0.522	4.59	236.34	400.97	1.105	2.11	0.698	42.19
MA	*O. fragrans*	2	138	0.006	0.096	0.254	0.166	0.414	7.39	129.56	251.32	0.445	1.52	0.557	30.64
MA	*O. fragrans*	3	272	0.005	0.139	0.231	0.288	0.466	6.39	94.69	102.19	0.551	2.17	0.575	27.12
TR	*O. fragrans*	1	227	0.005	0.172	0.342	0.257	0.326	3.36	286.05	57.69	0.283	1.73	0.367	27.80
TR	*O. fragrans*	2	188	0.006	0.111	0.321	0.133	0.348	4.04	154.55	30.69	0.430	3.27	0.316	26.83
TR	*O. fragrans*	3	236	0.006	0.145	0.263	0.140	0.398	4.48	228.95	40.32	0.234	1.17	0.424	25.48
GS	*O. fragrans*	1	244	0.006	0.192	0.088	0.180	0.684	6.42	116.36	124.85	0.605	1.97	0.625	27.48
GS	*O. fragrans*	2	269	0.002	0.183	0.094	0.274	0.726	8.16	149.03	176.94	1.645	1.33	0.794	34.34
GS	*O. fragrans*	3	243	0.004	0.162	0.089	0.252	0.633	6.93	138.61	161.96	0.676	1.57	0.721	31.66

WS	*O. fragrans*	1	236	0.026	0.361	0.203	0.157	0.606	4.78	39.38	68.36	0.537	8.00	0.565	32.81
WS	*O. fragrans*	2	145	0.008	0.227	0.198	0.380	0.430	4.99	75.48	31.32	0.650	3.31	0.362	25.66
WS	*O. fragrans*	3	265	0.019	0.334	0.158	0.239	0.644	5.50	42.74	55.56	0.657	9.45	0.559	32.89
WB	*O. fragrans*	1	198	0.007	0.092	0.085	0.168	0.336	4.84	16.29	155.17	0.249	0.61	0.368	19.68
WB	*O. fragrans*	2	248	0.007	0.139	0.119	0.248	0.522	3.68	90.93	212.46	0.782	2.73	0.584	17.95
WB	*O. fragrans*	3	207	0.006	0.144	0.097	0.226	0.478	4.79	120.92	215.62	0.814	3.05	0.455	18.93
HZ	Soil	1	11151	0.126	23.40	0.234	12.33	48.74	36.03	370.94	325.27	17.01	35.30	121.23	62.38
HZ	Soil	2	12600	0.103	21.52	0.387	12.30	48.41	28.90	391.99	309.18	19.10	36.91	121.16	92.54
HZ	Soil	3	14174	0.120	26.23	0.281	12.99	52.56	30.37	394.75	375.43	15.70	36.99	130.20	66.40
HX	Soil	1	18326	0.106	21.57	0.159	17.07	50.88	28.44	750.10	421.32	16.82	30.40	121.66	63.92
HX	Soil	2	15756	0.104	22.23	0.202	18.00	50.68	28.56	751.48	441.61	17.76	33.12	123.18	72.61
HX	Soil	3	16680	0.115	21.76	0.215	17.28	50.99	29.69	765.33	442.55	18.50	46.22	123.01	81.59
JX	Soil	1	14317	0.078	23.82	0.141	15.62	49.09	25.29	503.65	337.87	17.39	27.70	123.66	52.46
JX	Soil	2	13654	0.087	24.00	0.153	16.27	52.81	26.85	534.55	380.55	18.80	33.96	132.45	63.18
JX	Soil	3	12062	0.073	21.87	0.100	15.32	49.05	25.35	506.80	303.02	19.29	27.40	123.49	58.74
LY	Soil	1	20496	0.138	23.99	0.198	18.46	50.69	31.98	800.03	391.87	17.47	39.84	122.72	76.43
LY	Soil	2	17667	0.112	24.00	0.224	17.42	43.22	30.70	594.42	342.58	17.14	44.54	111.37	88.39
LY	Soil	3	17487	0.150	23.50	0.225	19.48	52.52	33.31	861.13	379.82	18.95	39.70	128.94	80.08
MA	Soil	1	11091	0.090	21.42	0.176	16.44	45.33	25.38	660.48	345.66	18.50	30.38	114.12	56.78
MA	Soil	2	13888	0.080	22.32	0.160	17.00	46.90	27.58	689.85	341.80	19.44	30.47	117.09	68.73
MA	Soil	3	10332	0.098	21.25	0.170	16.08	45.06	25.53	660.51	331.27	17.12	28.63	113.50	58.65
TR	Soil	1	11563	0.079	22.89	0.196	15.89	48.24	26.12	503.13	542.97	19.63	30.06	130.48	55.09
TR	Soil	2	12241	0.088	23.08	0.176	15.36	48.74	25.71	493.55	559.44	23.02	34.30	129.36	57.49
TR	Soil	3	10365	0.075	22.75	0.166	15.56	50.02	26.21	518.52	544.97	17.24	29.49	131.80	55.18
GS	Soil	1	12682	0.194	20.03	0.184	12.58	38.74	26.29	985.65	279.67	17.14	29.12	92.86	65.31
GS	Soil	2	15085	0.225	22.85	0.228	14.96	45.08	28.88	1075.22	377.33	16.43	33.48	109.42	73.30

GS	Soil	3	13997	0.230	21.66	0.230	14.61	43.27	27.25	1044.68	358.47	15.50	34.00	103.06	77.85
WS	Soil	1	11972	0.074	22.42	0.151	18.48	53.08	27.97	739.98	323.21	19.98	27.39	133.32	80.99
WS	Soil	2	17094	0.080	23.23	0.141	19.14	55.10	28.79	747.18	342.21	22.67	31.07	138.54	77.89
WS	Soil	3	18967	0.075	22.62	0.151	19.49	55.93	28.62	786.53	321.47	20.86	25.58	141.83	73.22
WB	Soil	1	15585	0.068	24.53	0.109	17.77	52.64	25.30	609.13	452.56	19.19	28.11	134.17	54.90
WB	Soil	2	12734	0.057	22.78	0.095	18.24	52.99	25.08	642.08	429.13	17.89	27.33	136.46	56.61
WB	Soil	3	11942	0.067	22.50	0.136	18.37	50.40	25.01	637.73	437.21	18.34	30.15	134.32	59.96

Table S2. Pearson's correlations of metals accumulation between plants and soil based on nine sampling sites.

Correlation		Ag	As	Cd	Co	Cr	Cu	Mn	Mo	Ni	Pb	V	Zn
H-S	Coefficient (*r*)	0.332	-0.567	0.384	0.707	0.168	0.040	0.941	0.101	0.218	-0.190	-0.016	-0.042
H-S	*P*	0.384	0.112	0.307	0.033	0.665	0.919	0.000	0.797	0.573	0.624	0.967	0.916
C-S	Coefficient (*r*)	-0.063	-0.193	0.377	0.220	0.082	0.232	-0.100	-0.578	-0.844	0.573	-0.106	0.468
C-S	*P*	0.873	0.619	0.317	0.569	0.834	0.548	0.798	0.103	0.004	0.107	0.787	0.204
O-S	Coefficient (*r*)	-0.474	-0.115	0.300	-0.491	-0.108	-0.118	-0.403	-0.481	-0.286	-0.129	-0.609	0.047
O-S	*P*	0.198	0.768	0.432	0.180	0.782	0.763	0.283	0.190	0.455	0.740	0.082	0.905

Note: H-S: correlation of *H. angustifolium* to soil; C-S: correlation of *C. bodinieri* to soil; O-S: correlation of *O. fragrans* to soil;
